# Factors Associated With Contraceptive Use Among Antenatal Care Clients With 3 or More Children at a Central Hospital in Burundi: A Cross-Sectional Study

**DOI:** 10.24248/EAHRJ-D-18-00012

**Published:** 2018-11-23

**Authors:** Sylvestre Bazikamwe, Prosper Niyongabo, Salvator Harerimana

**Affiliations:** a Department of Obstetrics and Gynaecology, Kamenge University Hospital; b Department of Research, Institut National de Santé Publique du Burundi, Bujumbura, Burundi

## Abstract

**Background:**

The fertility rate in Burundi has remained consistently high since the 1980s, while the prevalence of contraceptive use in the country (22%) has been among the lowest in Africa. Reasons for low contraception uptake in Burundi have not been adequately clarified.

This study aimed to identify factors associated with contraceptive use among pregnant women who had at least 3 healthy children and sought antenatal care services at an urban tertiary hospital in Burundi.

**Methods:**

Data were collected from antenatal clients with 3 or more children at Kamenge University Hospital. Data analysis included univariate and multivariate methods as well as multiple logistic regression analysis using SPSS, version 16.0.

**Results:**

We enrolled 255 women with a mean age of 32±4.5 years. The majority (n=232, 91.0%) of participants were urban residents with low incomes, and most (n=227, 89.0%) were educated to the primary school level or lower. The mean parity was 4.2±1.4, and most women had either 3 (n=120, 47.1%), 4 (n=66, 25.9%), or 5 (n=43, 16.9%) children; 26 (10%) participants had at least 6 children. Most (n=166, 65.1%) participants were part of couples who desired to have a final number of 4 to 6 children. About half (n=129, 50.6%) of the participants were able to name 1 or 2 benefits of contraception, and 105 (41.2%) participants mentioned 3 or 4 benefits of contraception. The most commonly reported benefit of contraceptive use was that it allows for improved maternal and child health. Low rates of contraceptive use were reported by participants with partners who worked as farmers, those citing fewer benefits of contraception, and those who relied on neighbours as their main source of information about contraception.

**Conclusion:**

Knowledge of the benefits of contraception was among the strongest determinants of contraceptive use in this population. Farmers and traders were less likely to use contraceptives than participants who were engaged in other types of work. Medical personnel were the most relied upon source of information about contraception, and the strongest predictor of contraceptive use was the personal opinion that contraception is acceptable.

## INTRODUCTION

Sustainable societal development is closely linked to the right balance between available resources and population size. In many low-income countries, particularly in sub-Saharan Africa, this balance continues to be elusive.^1^ High birth and fertility rates perpetuate the cycle of resource limitations and poverty.^2^ Strategies promoting widespread adoption of family planning and contraception methods have effectively decreased fertility rates and some of the associated negative consequences.^3–5^ Despite the implementation of health policies favouring and promoting birth control, the synthetic fertility rate in Burundi was still 5.5 children per woman in 2016, marginally down from 6.8 in the 1980s and 6.4 in 2010.^6,7^ High fertility is strongly associated with high maternal morbidity and mortality rates.^3,4,8^ Burundi's high fertility rate certainly contributes to the high maternal mortality rate (392 deaths per 100,000 live births), and it has been linked to the country's high neonatal mortality rate (23 per 1,000 live births) as well as the high rate of obstetrical complications.^6,9^

The contraceptive use rate in Burundi among women in union is 29%, which is the lowest in the East African Community.^6^ According to the United Nations Development Programme's Vision Burundi 2025 project estimates, to achieve control of the country's current population growth rate, couples should not exceed having 3 living children.^10^ However, 32% of Burundian women with 3 children still desire more children in the near future.^6^ This could be a major obstacle towards achieving the Vision Burundi 2025's objectives related to population control.

To guide policies supporting Vision Burundi 2025, we attempted to identify factors associated with contraceptive use among pregnant women with at least 3 children.

## METHODS

### Study Design and Variables

This cross-sectional study was carried out between 8 December 2014 and 6 February 2015 to identify the factors influencing contraceptive use among Burundian women with 3 or more living children.

Data collection included participant sociodemographic characteristics and perceptions about contraceptive use and contextual factors, such as Burundi's political and institutional climate. The dependent variable was contraceptive use.

### Study Site, Population, and Participants

The study was conducted within the confines of the antenatal care service of Kamenge University Hospital, which is a 421-bed tertiary referral facility in Bujumbura, Burundi. The antenatal service, in the obstetrics and gynaecology department, manages about 9,950 women per year.

The study population consisted of consenting pregnant antenatal clients who had at least 3 children reported to be in good health. This population was targeted because of the likely substantial contribution to the country's high fertility rate by women who desire bearing additional children despite already having given birth to at least 3 healthy children. Any strategy aiming to slow down population growth should consider this segment of the population.

The sample size was calculated using Fisher's formula for cross-sectional studies,^11^ as follows:

$$N=\frac{z^{2}pq}{d^{2}}$$

where z=1.96 for the 95% confidence level; p=proportion of pregnant women utilising antenatal services at Kamenge University Hospital who have at least 3 children; q=(1 p); d=study precision (set at 0.05 for the 95% confidence level).

The proportion of antenatal care clients who had 3 or more living children was calculated using figures found in hospital registers. Owing to limitations in our medical record keeping capacity, this proportion was calculated only for 8 months, between 3 November 2012 and 8 July 2013. During that period, out of 809 pregnant women who utilised antenatal services at Kamenge University Hospital, 169 (20.9%) had at least 3 living children.

**FIGURE:**
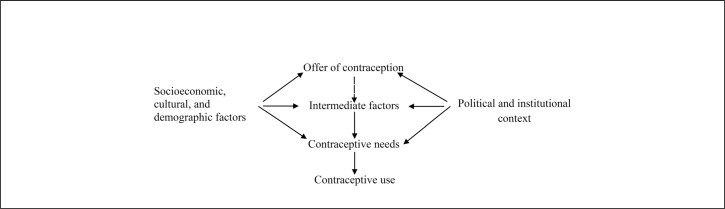
Conceptual Model of Determinants of Contraceptive Use^12^

The sample size calculation determined that we needed to enrol 255 pregnant women with at least 3 children. The first participant was randomly selected using a random number generator. Subsequently, we attempted to enrol every third antenatal client (based on the total population divided by the sample size, 809/255) seeking care at our centre until we reached the desired sample size.

### Conceptual Model

The study drew from the model of contraceptive use proposed by Akam et al^12^ in their study about contraceptive use in Cameroon (**Figure**). This model depicts factors that potentially determine the use of contraceptives within a given population and the links between these factors. We also assessed the influence of knowledge among participants regarding the benefits of contraception using a tool developed by Singh.^8^ Topics addressed by the tool include maternal and child health improvement, increasing household wealth, prevention of obstetrical complications, and children's educational opportunities. A score was calculated for every participant according to their knowledge about the benefits of contraception.

### Data Collection and Analysis

Data were collected, using a structured questionnaire, by a female medical assistant trained in quantitative research methods. The questionnaire was pretested before its formal use, and adjustments were made to ensure its reliability and validity. Data analysis included descriptive statistics using frequencies, percentages, and means. Thereafter, univariate analysis was done between each potential determinant and the dependent variable, and statistical significance was determined using the chi-square test.

Multivariate analysis based on adjusted odds ratios and multiple logistic regression were used to assess the strength of the relationships between variables in the final best fit model with 95% confidence intervals (CIs). Data analysis was carried out using SPSS, version 16.0 (SPSS Inc., Chicago, IL, USA).

### Ethical Considerations

The study was officially approved by the National Ethical Committee in October 2014. Codes were assigned to client files to ensure anonymity.

## RESULTS

### Participant Characteristics

#### Sociodemographic Characteristics

The mean age of the participants was 32±4.5 years (range, 21–44 years). Most participants were urban residents (n=232, 91.0%) with low incomes and primary-level education or less (n=227, 89.0%).

##### Parity and Number of Children

The mean parity was 4.261.4 (range, 3–10 deliveries). Most women had either 3 (n=120, 47.1%), 4 (n=66, 25.9%), or 5 (n=43, 16.9%) children; 26 (10%) participants had at least 6 children (**Table 1**).

**TABLE 1.** Number of Living Biological Children Among Participants

Number of Children	n (%)
3	120 (47.1)
4	66 (25.9)
5	43 (16.9)
6	13 (10.2)
7	7 (2.7)
8	4 (1.6)
9	2 (0.8)
**Total**	**255 (100)**

### Participants' Knowledge of the Benefits of Contraception

Participants were asked to mention some benefits of contraceptive use, and the following were the expected responses: contraception leads to (1) improvement of the national economy, (2) better educational opportunities for children, (3) prevention of obstetrical complications, (4) improvement of family finances, (5) improvement of maternal health, (6) and improvement of children's health. Each participant was assigned a score based on the number of benefits she was able to list (**Table 2**). Regarding knowledge of the benefits of contraception, most women achieved a score of 1 (1 or 2 benefits mentioned; n=129, 50.6%) or 2 (3 or 4 benefits mentioned; n=105, 41.2%).

**TABLE 2.** Number of Benefits of Contraception Reported by Participants

Score	Interpretation	n (%)
0	No benefits reported	11 (4.3)
1	1–2 benefits reported	129 (50.6)
2	3–4 benefits reported	105 (41.2)
3	5–6 benefits reported	10 (3.9)

### Contraceptive Use Among Study Participants

Ninety-eight (38.4%) participants reported having never used contraception, 157 (61.6%) had interrupted contraception before the current pregnancy, and 37 (14.5%) were opposed to contraception.

Rates of reported contraceptive use were highest among women aged 30 to 34 years (76 of 106, 71.7%) and those aged 40 to 44 years (6 of 8, 75.0%), and contraceptive use was reportedly lowest among participants aged 20 to 24 years (4 of 9, 44.4%) (**Table 3**). Reported contraceptive use was relatively high among women with a parity of 4 or 5 (79 of 120, 65.8%) and low among participants wishing to have 7 or more children (7 of 15, 46.7%).

**TABLE 3.** Factors Significantly Associated With Contraceptive Use

	Reported Contraceptive Use Before Current Pregnancy		
Variables	No n (%)	Yes n (%)	*P* Value
**Age (years)**			
20–24	5 (55.6)	4 (44.4)	.020
25–29	28 (52.8)	25 (47.2)	
30–34	30 (28.3)	76 (71.7)	
35–39	33 (41.8)	46 (58.2)	
40–44	2 (25.0)	6 (75.0)	
**Parity**			
3	34 (35.8)	61 (64.2)	.025
4–5	41 (34.2)	79 (65.8)	
≥6	23 (57.5)	17 (42.5)	
**Desired final number of children**			
1–3	5 (35.7)	9 (64.3)	.026
4–6	53 (32.1)	112 (67.9)	
≥7	8 (53.3)	7 (46.7)	
Undetermined	32 (52.5)	29 (47.5)	
**Knowledge of benefits of contraception**			
No benefits reported	9 (81.2)	2 (18.2)	.025
1–2 benefits reported	45 (34.9)	84 (65.1)	
3–4 benefits reported	40 (38.1)	65 (61.9)	
5–6 benefits reported	4 (40.0)	6 (60.0)	
**Main source of information**			
Medical personnel	58 (27.4)	154 (72.6)	<.001
Church	4 (80.0)	1 (20.0)	
Neighbours	31 (96.9)	1 (3.1)	
Radio/television	5 (83.3)	1 (16.7)	
**Opinion on contraception**			
Not acceptable	34 (91.9)	3 (8.1)	<.001
Acceptable	64 (29.4)	154 (70.6)	

Participants with less knowledge of the benefits of contraception were less likely to report contraceptive use (**Table 3** and **Table 5**). Additionally, participants who reported personal acceptance of contraception reported contraceptive use significantly more often than those who were opposed to contraceptive use (adjusted odds ratio 43.5; 95% CI, 10.7 to 177; *P*<.001).

**TABLE 4.** Factors Not Significantly Associated With Contraceptive Use

	Reported Contraceptive Use Before Current Pregnancy		
Variables	No n (%)	Yes n (%)	*P* Value
**Occupation**			
Farmer	47 (40.2)	70 (59.8)	.211
Employed	9 (42.9)	12 (57.1)	
Traders	26 (44.8)	32 (55.2)	
Other	16 (27.1)	43 (72.9)	
**Partner's occupation**			
Farmer	33 (49.3)	34 (50.7)	.074
Employed	17 (41.5)	24 (58.5)	
Trader	19 (43.2)	25 (56.8)	
Driver	13 (27.1)	35 (72.9)	
Other	16 (29.1)	39 (70.9)	
**Level of education**			
Less than primary	42 (38.2)	68 (61.8)	.552
Primary	44 (37.6)	73 (62.4)	
Secondary	8 (36.4)	14 (63.6)	
Tertiary	4 (66.7)	2 (33.3)	
**Partner's level of education**			
Less than primary	29 (39.7)	44 (60.3)	.118
Primary	50 (35.5)	91 (64.5)	
Secondary	10 (35.7)	18 (64.3)	
Tertiary	9 (69.2)	4 (30.8)	
**Marital status**			
Married	71 (39.4)	109 (60.6)	.806
Separated	1 (50.0)	1 (50.0)	
Widow	0 (0.0)	1 (100)	
Free union	26 (36.1)	46 (63.9)	
**Religion**			
Catholic	30 (35.7)	54 (64.3)	.764
Christian, non-Catholic	61 (40.4)	90 (59.6)	
Muslim	7 (36.8)	12 (63.2)	
Other	98 (38.4)	157 (61.6)	

**TABLE 5.** Logistic Regression Results for Determinants of Contraceptive Use (N=255)

Variables	n (%)	AOR (95% CI)	*P* Value
**Partner's occupation**			
Farmer	67 (26.2)	6.82 (2.15–21.5)	.001
Employed	41 (16.1)	3.57 (1.05–12.0)	.041
Trader	44 (17.2)	5.85 (1.62–21.1)	.007
Mechanic agent	48 (18.8)	2.59 (0.71–9.39)	.147
Other	55 (21.5)	1	
**Knowledge of benefits of contraception**			
No benefits reported	11 (4.3)	8.55 (0.96–75.8)	.054
1–2 benefits reported	129 (50.6)	0.44 (0.10–1.91)	.277
3–4 benefits reported	105 (41.2)	0.69 (0.16–2.85)	.611
5–6 benefits reported	10 (3.9)	1	
**Main source of information**			
Medical personnel	212 (83.1)	0.10 (0.02–0.45)	.003
Church	5 (2.0)	0.35 (0.01–7.84)	.512
Neighbours	32 (12.5)	14.6 (1.23–173)	.033
Radio/television	6 (2.4)	1	
**Opinion on contraception**			
Not acceptable	37 (14.5)	43.5 (10.7–177)	<.001
Acceptable	218 (85.5)	1	

Abbreviations: AOR, adjusted odds ratio; CI, confidence interval

Four variables were significantly associated with the use of contraceptives in the final logistic regression model: partner's occupation, knowledge score regarding the benefits of contraception, the main source of information on contraception, and contraception acceptance (**Table 5**).

## DISCUSSION

Contraception remains the most effective strategy to reduce maternal and neonatal mortality in developing countries, particularly in sub-Saharan Africa.^4^ It is also 1 of the 4 pillars of the Safe Motherhood initiative. Most of the determinants of contraceptive use investigated in this study have also been investigated in other developing countries.^12–15^

Knowledge of the advantages of contraception is among the main factors leading to its use,^8^ but as the majority of our participants were educated to the primary school level or lower, the lack of knowledge about the benefits of contraceptive use might not be surprising. Similar observations were made in a study investigating high fertility and low contraceptive use among young people in Uganda.^16^

Participants who worked as farmers and traders were less likely to use contraception in our study, compared with the other employment categories. Additionally, women whose partners were farmers or traders were, respectively, 7 and 6 times less likely to use contraceptives than women with partners in other occupations. Other studies carried out in low- and middle-income countries have established that women with lower levels of formal education and those in the lower and middle social classes are less likely to use contraception than those from high-income households or backgrounds.^17–21^ Low literacy rates, poor access to information, and poor health infrastructure are particularly widespread in sub-Saharan Africa. Moreover, it has been reported that traditional African society is structured in such a way that high fertility and large surviving families are often considered economically and socially rewarding, in contrast with modern societies elsewhere.^1,20^

Information sources also play a role in contraception uptake.^22–24^ In our study, medical personnel were the most frequently reported source of information about contraception, over neighbours, radio or television, and the Church. In rural Malawi, it has been shown that the media can play a significant role in improving maternal health outcomes when it is community-led and locally driven.^22^

Acceptance of the practice of contraception was the strongest predictor of contraceptive use in our analysis. Approval or disapproval of contraception has previously been reported to be strongly influenced by religion in Burundi; we, therefore, recommend that political authorities and health-care leaders consider prioritising reproductive health issues, including contraception, in their correspondence and interactions with Burundian religious leaders.^25^

### Limitations

This study did not assess participants' knowledge about contraceptive methods, which could have enriched our findings. However, the identified factors provide sufficient scientific value and can be used to inform policy discussions and awareness campaign planning, for example. Moreover, we did not assess men's opinions on contraception or the influence of side effects on contraceptive use in our study population, as we thought that these issues would be better investigated using qualitative methods.

## CONCLUSION

This study has contributed to a better understanding of contraceptive use among multiparous women in the study area. Knowledge of the benefits of contraception was among the main factors leading to contraceptive use. Farmers and traders were less likely to use contraception compared with individuals earning a living through other types of work. Medical personnel were the most commonly sought source of information on contraception. Personal acceptance of the practice of contraception was the strongest predictor of contraceptive use. Health policy managers could use these findings to guide interventions promoting contraceptive use.

## References

[1] Caldwell JC, Caldwell P. The cultural context of high fertility in sub-Saharan Africa. Popul Dev Rev. 1987;13(3):409–437. 10.2307/1973133

[2] Barot S. Back to basics: the rationale for increased funds for international family planning. Guttmacher Policy Rev. 2008;11(3):13–18.

[3] Stover J, Ross J. How increased contraceptive use has reduced maternal mortality. Matern Child Health J. 2010;14(5):687–695. 10.1007/s10995-009-0505-y. Medline 19644742

[4] Ahmed S, Li Q, Liu L, Tsui AO. Maternal deaths averted by contraceptive use: an analysis of 172 countries. Lancet. 2012;380(9837):111–125. 10.1016/S0140-6736(12)60478-4. Medline 22784531

[5] Letamo G, Letamo HN. The role of proximate determinants in fertility transition: a comparative study of Botswana, Zambia, and Zimbabwe. South Afr J Demogr. 2001-2002;8(1):29–35.

[6] Ministère à la Présidence chargé de la Bonne Gouvernance et du Plan [Burundi] (MPBGP), Ministère de la Santé Publique et de la Lutte Contre le SIDA [Burundi] (MSPLS), Institut de Statistiques et d'Études Économiques du Burundi (ISTEEBU), ICF. Troisième Enquête Démographique et de Santé au Burundi 2016-2017. Bujumbura, Burundi: ISTEEBU, MSPLS, and ICF; 2017. https://dhsprogram.com/pubs/pdf/FR335/FR335.pdf. Accessed on 27 October 2018.

[7] Ministère de l'Intérieur Département de la Population [Burundi] (MIDP), Institute for Resource and Development (IRD). Enquête Démographiqueet de Santé au Burundi 1987. Gitega, Burundi, and Columbia, MD, USA: MIDP and IRD; 1988. https://www.dhsprogram.com/pubs/pdf/FR6/FR6.pdf. Accessed 27 October 2018.

[8] Singh S, Darroch J, Vlassoff M. Adding It Up: The Costs and Benefits of Investing in Family Planning and Maternal and Newborn Health. New York: Guttmacher Institute and United Nations Population Fund; 2009. https://www.guttmacher.org/sites/default/files/report_pdf/AddingItUp2009.pdf. Accessed 27 October 2018.

[9] Ministère de la Santé Publique et de la Lutte Contre le SIDA (Burundi) (MSPLS). Evaluation des Besoins en Matiere de Soins Obstetricaux et Neonatals d'Urgence au Burundi (EB SONU) – Rapport Définitif. Bujumbura, Burundi: MSPLS; 2011. https://www.minisante.bi/images/EB%20SONU.pdf. Accessed 27 October 2018.

[10] Institut de Statistiques et d'Études Économiques du Burundi (ISTEEBU), United Nations Population Fund (UNFPA). La CIPD Après 2014: Rapport National. Bujumbura, Burundi: ISTEEBU and UNFPA; 2014. https://burundi.unfpa.org/sites/default/files/resource-pdf/rapportcipd2014.pdf. Accessed 27 October 2018.

[11] Fisher LD. Self-designing clinical trials. Stat Med. 1998;17(14):1551–1562. 10.1002/(SICI)1097-0258(19980730)17:14<1551::AID-SIM868>3.0.CO;2-E. Medline 9699229

[12] Akam E, Ngoy. Utilisation des méthodes contraceptives en Afrique: de l'espacement à la limitation des naissances? In: Gendreau F, Poupard M, editors. Les Transitions Démographiques des Pays du Sud. Paris: Éditions ESTEM; 2001. http://horizon.documentation.ird.fr/exl-doc/pleins_textes/divers17-09/010027446.pdf. Accessed 27 October 2018.

[13] Issa Z. Facteurs Associés à la Non-utilisation de la Contraception Moderne Chez les Femmes en Union Dans la Partie Septentrionale du Cameroun [dissertation]. Yaounde, Cameroon: Université de Yaounde II; 2008.

[14] Sedgh G, Hussain R. Reasons for contraceptive nonuse among women having unmet need for contraception in developing countries. Stud Fam Plann. 2014;45(2):151–169. 10.1111/j.1728-4465.2014.00382.x. Medline 24931073

[15] Peer N, Morojele N, London L. Factors associated with contraceptive use in a rural area in Western Cape Province. S Afr Med J. 2013;103(6):406–412. 10.7196/SAMJ.6201. Medline 23725962

[16] Nalwadda G, Mirembe F, Byamugisha J, Faxelid E. Persistent high fertility in Uganda: young people recount obstacles and enabling factors to use of contraceptives. BMC Public Health. 2010;10(1):530. 10.1186/1471-2458-10-530. Medline 20813069 PMC2940919

[17] Dwivedi SN, Sundaram KR. Epidemiological models and related simulation results for understanding of contraceptive adoption in India. Int J Epidemiol. 2000;29 (2):300–307. 10.1093/ije/29.2.300. Medline 10817129

[18] Ali MM. Quality of care and contraceptive pill discontinuation in rural Egypt. J Biosoc Sci. 2001;33(2):161–172. 10.1017/S0021932001001614. Medline 11284624

[19] Bentley R, Kavanagh A, Smith A. Area disadvantage, socioeconomic position and women's contraception use: a multilevel study in the UK. J Fam Plann Reprod Health Care. 2009;35(4):221–226. 10.1783/147118909789587277. Medline 19849915

[20] Palamuleni ME. Demographic and socioeconomic factors affecting contraceptive use in Malawi. J Hum Ecol. 2014;46(3):331–341. 10.1080/09709274.2014.11906731

[21] Todd CS, Isley MM, Ahmadzai M, et al. Cross-sectional analysis of factors associated with prior contraceptive use among hospitalized obstetric patients in Kabul, Afghanistan. Contraception. 2008;78(3):249–256. 10.1016/j.contraception.2008.05.005. Medline 18692617 PMC2585412

[22] Zamawe COF, Banda M, Dube AN. The impact of a community driven mass media campaign on the utilisation of maternal health care services in rural Malawi. BMC Pregnancy Childbirth. 2016;16:21. 10.1186/s12884-016-0816-0. Medline 26819242 PMC4730729

[23] Afolabi BM, Ezedinachi EN, Arikpo I, et al. Knowledge, non-use, use and source of information on contraceptive methods among women in various stages ofreproductive age in rural Lagos, Southwest Nigeria. Open Access J of Contracept. 2015;6:65–75. 10.2147/oajc.s80683. Medline 29386924 PMC5683143

[24] Japaridze T, Kristesashvili J, Imnadze P. The influence of sources of information on contraception use in Georgia. Georgian Med News. 2015;248(11):16–20. Medline 26656545

[25] Niyongabo P, Douwes R, Dieleman M, et al. Ways and channels for voice regarding perceptions of maternal health care services within the communities of the Makamba and Kayanza provinces in the Republic of Burundi: an exploratory study. BMC Health Serv Res. 2018;18:46. 10.1186/s12913-017-2822-y. Medline 29378564 PMC5789700

